# Diagnostic performance of a wearing dynamic ECG recorder for atrial fibrillation screening: the HUAMI heart study

**DOI:** 10.1186/s12872-021-02363-1

**Published:** 2021-11-20

**Authors:** Wenxia Fu, Ruogu Li

**Affiliations:** Department of Cardiac Function, Shanghai Chest Hospital, Shanghai Jiao Tong University, 241 Huaihai West Rd, Xuhui District, 200030 Shanghai, China

**Keywords:** Atrial fibrillation, Detection, Accuracy, Dynamic ECG recorder, One-lead ECG

## Abstract

**Background:**

Atrial fibrillation (AF) is the most prevalent cardiac dysrhythmia with high morbidity and mortality rate. Evidence shows that in every three patients with AF, one is asymptomatic. The asymptomatic and paroxysmal nature of AF is the reason for unsatisfactory and delayed detection using traditional instruments. Research indicates that wearing a dynamic electrocardiogram (ECG) recorder can guide accurate and safe analysis, interpretation, and distinction of AF from normal sinus rhythm. This is also achievable in an upright position and after exercises, assisted by an artificial intelligence (AI) algorithm.

**Methods:**

This study enrolled 114 participants from the outpatient registry of our institution from June 24, 2020 to July 24, 2020. Participants were tested with a wearable dynamic ECG recorder and 12-lead ECG in a supine, an upright position and after exercises for 60 s.

**Results:**

Of the 114 subjects enrolled in the study, 61 had normal sinus rhythm and 53 had AF. The number of cases that could not be determined by the wristband of dynamic ECG recorder was two, one and one respectively. Case results that were not clinically objective were defined as false-negative or false-positive. Results for diagnostic accuracy, sensitivity, and specificity tested by wearable dynamic ECG recorders in a supine position were 94.74% (95% CI% 88.76–97.80%), 88.68% (95% CI 77.06–95.07%), and 100% (95% CI 92.91–100%), respectively. Meanwhile, the diagnostic accuracy, sensitivity and specificity in an upright position were 97.37% (95% CI 92.21–99.44%), 94.34% (95% CI 84.03–98.65%), and 100% (95% CI 92.91–100%), respectively. Similar results as those of the upright position were obtained after exercise.

**Conclusion:**

The widely accessible wearable dynamic ECG recorder integrated with an AI algorithm can efficiently detect AF in different postures and after exercises. As such, this tool holds great promise as a useful and user-friendly screening method for timely AF diagnosis in at-risk individuals.

## Background

Atrial fibrillation (AF) is the most common cardiac dysrhythmia, with an estimated prevalence rate of 2% and 4% in adults [1]. Emerging evidence indicates that the incidence of AF substantially increases with age (6%; > 65-years of age and 8–15%; > 80-years of age) [2, 3]. Moreover, findings of the ATRIA study projected a possibility that nearly 3 million individuals aged above 80-years will be infected with AF by 2050 [4]. AF is related to significant morbidity and mortality, therefore remains a major public health threat [5]. The 2020 ESC guidelines suggested that the lifetime risk of AF increased from one in four individuals of European descent to one in three individuals by age 55 [6]. AF is primarily associated with an increased risk of stroke and heart failure, thereby significantly impact people’s quality of life and longevity [7, 8]. More evidence shows that AF increases the risk of stroke by approximately five times higher [5] and results in a two-fold risk of heart failure [9].

A previous investigation by Stewart et al. showed that AF constitutes a significant prevalent ratio in the global burden of disease and accounts for 1% of Britain’s National Health Service budget [10]. Elsewhere, Kim et al. revealed that AF contributed significantly to the US Net expenditure. approximated at $16 billion and $26 billion per annum [11]. Emerging data indicate that, of three patients with AF, one is asymptomatic [12]. Hence, a majority of individuals develop heart failure or stroke as the first symptom of AF. Strikingly, asymptomatic AF carries the same risks and sometimes even has more adverse outcomes compared to symptomatic AF [13]. Challenges with the detection of asymptomatic AF in the early stages are becoming an increasing concern. Besides, the European guidelines recommend opportunistic screening for patients with AF over 65-years of age, including high-risk patients [14].

The asymptomatic and paroxysmal nature of AF may result in unsatisfactory and delayed detection using traditional instruments, such as the gold-standard 12-lead electrocardiogram (ECG) [15]. Significantly, ECG is subject to testing space and time limitations and can only be examined in a hospital. In this view, Holter monitoring is associated with prolonged ECG monitoring and makes up for the time limitation caused by 12-lead ECG. Although Holter improves the detection rate of paroxysmal AF, long-term monitoring (24-h and 72-h) influences the patients’ daily routine. For example, some patients complain of skin irritation [16].

Notwithstanding cost and other factors, including the inconvenience it causes to patients, large-scale population screening is nearly impossible. The present investigation focused on the patient’s resting position without reflecting on the state of the real world. Given these, a rational and user-friendly approach is warranted for early screening and detection of AF in different states. The recent past has seen the increasing use of wristbands as a health management tool. Several individuals wear wristbands daily. Therefore, wearing dynamic ECG recorders will guide the distinction of AF from normal sinus rhythm, accurately and safely in the Chinese population irrespective of postural states.

## Methods

### Study population

**Fig. 1:**
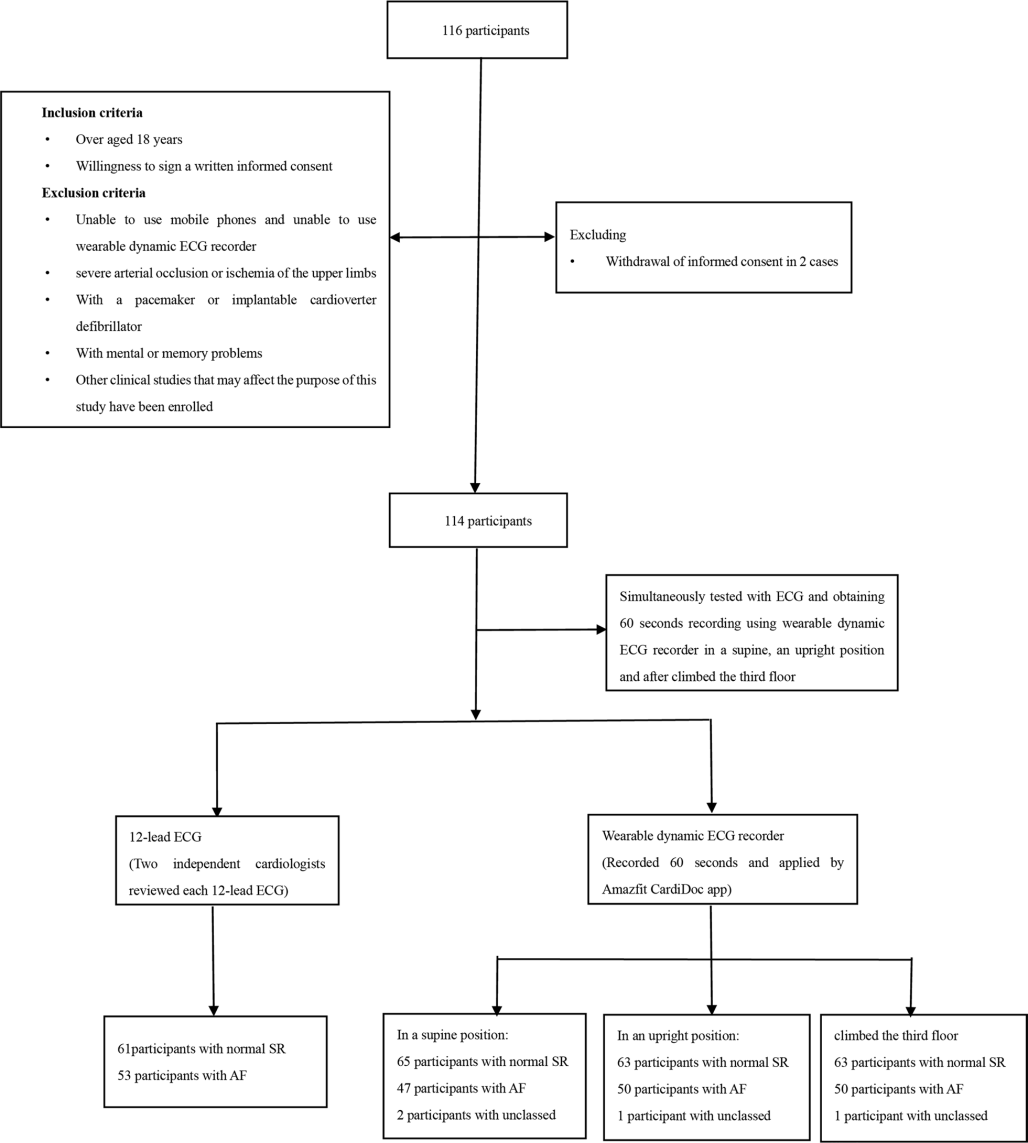
A flowchart of the study. *AF* atrial fibrillation, *ECG* electrocardiogram, *SR* sinus rhythm

A total of 116 outpatients were recruited from the Shanghai Chest Hospital between June 24, 2020 and July 24, 2020. Two participants withdrew informed consent halfway through the study. As such, 114 participants met the inclusion criteria for participation in the study. Participants were aged 18-years and older. Exclusion criteria: (i) Participants who were unable to hold both devices; (ii) participants with severe arterial occlusion or ischemia of the upper limbs; (iii) patients with a pacemaker or implantable cardioverter-defibrillator; (iv) patients who registered for other clinical studies that could impact the purpose of the study. Patients diagnosed as “AF” by 12-lead ECG were classified as AF positive, whereas and AF negative patients served as AF control groups. Standardized in-person interviews per conducted to collect data on baseline characteristics, including demographics, medical history, and medications. A flowchart of the study is shown in Fig. 1.

This is a prospective, registration-only, single-center study whose overall goal is to assess the effectiveness and safety of AF detection and provide a reliable non-invasive method for screening and management of AF in daily routines (ClinicalTrials.govNCT04462653). The local ethics committee of Shanghai Chest Hospital approved this research (No.LS2035), and all participants gave written informed consent before participation in the study. The study was performed following the Helsinki Declaration. The wearable dynamic ECG recorder was approved by the China Food and Drug Administration (Anhui Device Registration Approval No. 20182210012).

### Signal acquisition and processing

Each participant was asked to lie down in a supine position. The choice of an individual’s left-or right-hand wristband is pre-set in the wristband’s application. Participants held the wearable dynamic ECG recorder on the left wrist on the bed in the ECG room to ensure consistency of results. Of note, during the tests, the attending physician made sure each participant touched the button attached to the 6 o’clock side of the wearable dynamic ECG recorder. After getting tested with a 12-lead ECG for 60 s, subjects actively triggered the wearable dynamic ECG recorder to record the ECG signal for 60 s. Next, participants were asked to wear the wristband in an upright position for 60 s and after that, they climbed to the third floor and re-tested for 60 s. Finally, a full 12-lead ECG recording for 15 s was performed immediately to confirm the diagnosis of ECG at this point.

Furthermore, participants were asked to rest their right forefinger on the touch button and press their right forefinger using sufficient force to ensure that the contact of electrode sensors with their left wrist’s skin for the entire recording duration. Data acquired when a participant wore the wristband on the left hand with the right thumb on the touch button was seen to be equivalent to limb Lead I. The ECG data was transmitted to the Amazfit CardiDoc application and Cloud servers in real-time and analyzed via artificial intelligence (AI) algorithm.

**Fig. 2:**
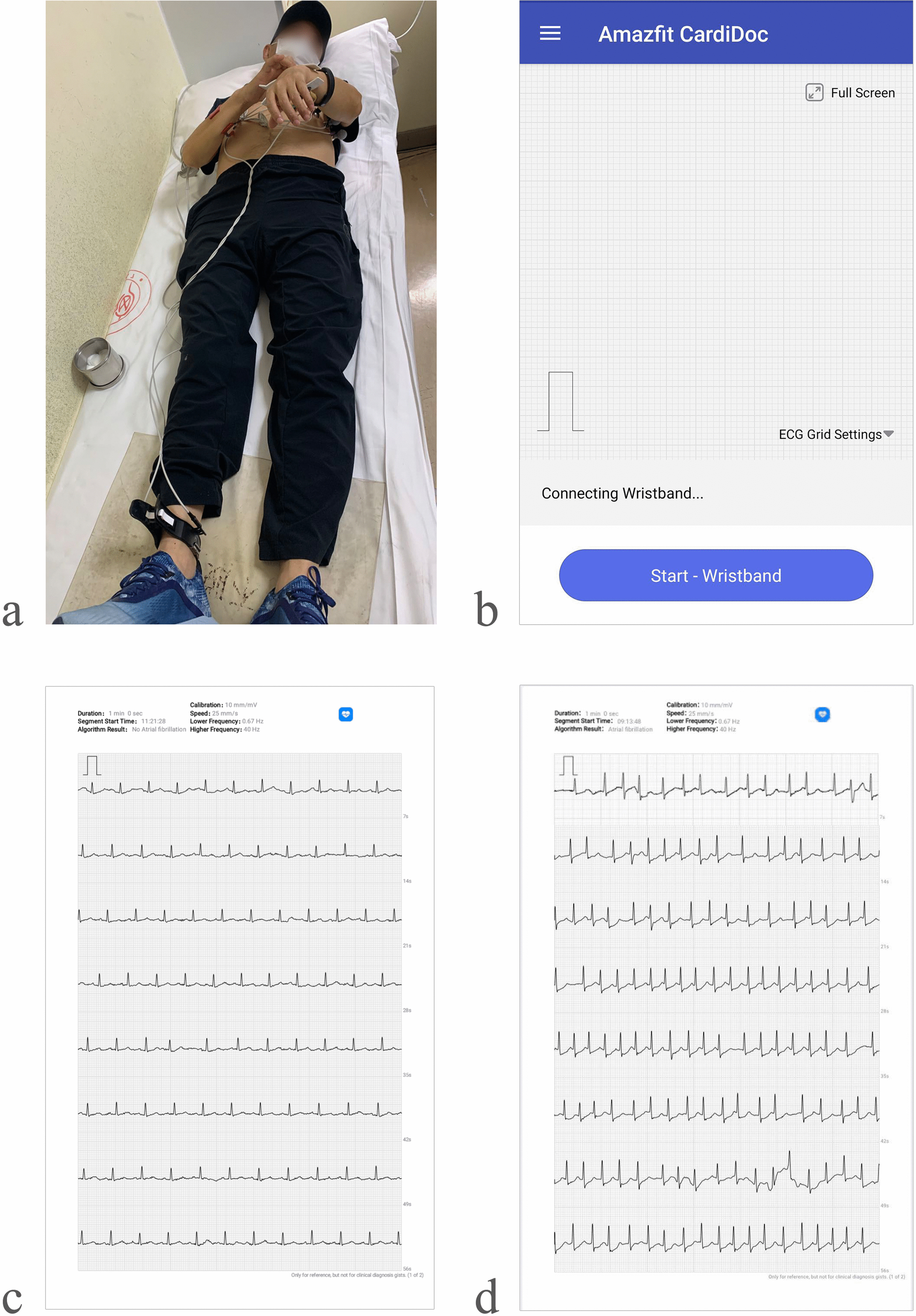
A prototype for AF detection using wearable dynamic ECG recorder and 12-lead ECG. **A** A patient is simultaneously tested with a wearable dynamic ECG recorder and 12-lead ECG. **B** A screenshot of the Amazfit CardiDoc app installed on the mobile phone. **C** Representative pulse waveform recording a participant with normal sinus rhythm. **D** Representative pulse waveform recording a participant with AF. *AF* atrial fibrillation, *ECG* electrocardiogram

The device comprised a wristband, ECG recorder, the embedded software (installed on the ECG recorder), mobile phone application (Amazfit CardiDoc application installed on the mobile phone, which transmits the ECG data over the network to the cloud servers), and charging cable, designed to capture the ECG data of the user and transmit it via Bluetooth to an iOS or Android mobile phone. The AmazfitCardiDoc application can store thousands of recordings on the mobile phone. These recordings are accessible to authorized users on cloud servers. The application determines the presence or absence of a classified waveform for AF or sinus rhythm, which is not recommended for other users with known arrhythmias. Figure 2 shows a prototype for AF detection using the wearable dynamic ECG recorder and 12-lead ECG.

### Rhythm analysis

12-lead ECG recordings served as the reference standard for heart rhythm classification. Two independent electrophysiologists who were blinded to the analysis of results reviewed the 12-lead ECG. Possible disagreements were solved by consensus. The software would automatically save the ECG data immediately after the wearable dynamic ECG recorder recordings were complete. The delivered diagnosis was cited by an AI algorithm either as “non-AF,” “AF,” and “unclassified.” If the signal quality was too poor, unstable, or the requirements were not met, the output was “unclassified.” The recording stopped if the contact with the electrode was disconnected during the acquisition, therefore was restarted.

### Study outcomes

The primary outcome of the study was the wearable dynamic ECG accuracy of the recorder compared to the 12-lead ECG in detecting AF. The secondary outcomes were the sensitivity, specificity, positive predictive value (PPV), and negative predictive value (NPV) of the wearable dynamic ECG recorder in detecting AF. Safety evaluation criteria included the occurrence of adverse events (AE) and device defects. AE and serious adverse events (SAE) during the clinical study were recorded, and the potential association of AE and SAE with the test device was determined. Device defects, including problems associated with the use of test devices, such as failure to wear, no result output, and signal interference were noted.

### Statistical methods

Usually, a sample size of 108 outpatients is required with an 80% power and an *α* of 0.05. Herein, considering a roughly 5% dropout rate, six outpatients were needed to ensure complete data. Continuous variables with a normal distribution were presented as mean ± standard deviation (SD). Dates with discrete variables were presented as percentages. Besides, dates with non-normal distribution were presented as medians and interquartile ranges. The estimated 30% of patients with AF, 54 positive AF samples, and 60 non-AF samples were selected.

Data were analyzed by t-test, Chi-square, or Fisher’s exact tests, and the Wilcoxon tests as appropriate. A four-fold table was constructed to diagnose “AF” or “non-AF” using a 12-lead ECG and “AF” or “non-AF” using a wristband algorithm. During AF diagnosis, the matching rate of the difference between the AI algorithm of wristband judgment and human judgment assisted in the evaluation of the consistency of positive results. The number of true positives (TP), true negatives (TN), false positives (FP), and false negative (FN) from the AF and typical sinus rhythm databases were counted.

Subsequently, the sensitivity TP/(TP + FN), specificity TN/(TN + FP), positive predictive value true positive TP/(TP + FP), negative predictive value TN/(FN + TN), and accuracy (TP + TN)/(TP + TN + FP + FN) were calculated. The “unclassified” cases were defined as the true positive and true negative or false positive and false negative, and then counted and described separately. Statistical analysis was performed by SPSS 22.0 software for Windows (IBM Corp., Armonk, NY, USA). A *P-*value of < 0.05 denoted statistical significance.

## Results

### Study population and baseline characteristics

A total of 114 subjects (61 with normal sinus rhythm and 53 with AF), 65 males and 49 females, mean age 59 ± 11.16 years (range: 25–80 years), average height 1.66 ± 0.08 m (range: 1.50–1.80 m), average weight 66.1 ± 11.96 kg (range: 40–105 kg), average body mass index 23.87 ± 3.28 kg/m^2^ (range: 16.65–33.75 kg/m^2^) were included in the study. Compared to the non-AF group, subjects in the AF group were: (i) significantly older (*P* < 0.001); (ii) had a higher proportion of participants with coronary heart disease (*P* = 0.014); (ii) had a higher thromboembolic risk based on the CHA2DS2VASc (*P* = 0.001); (iv) used oral anticoagulants, antiplatelet agents, calcium channel blockers (CCB), diuretics, digoxin, and β-blockers at significantly higher rates (All *P* < 0.05) (Table 1).

**Table 1:** Baseline demographic of in Non-AF and AF group (N = 114)

Variable	Non-AF (n = 61)	AF (n = 53)	*P-*value
Demographics			
Age (years)	55.15 ± 11.01	64.00 ± 9.38	< 0.001
Females (n, %)	25 (41.0)	24 (45.3)	0.644
Height (m)	1.66 ± 0.08	1.66 ± 0.08	0.619
Weight (kg)	66.16 ± 11.89	66.07 ± 12.15	0.965
BMI (kg/m^2^)	23.97 ± 3.21	23.74 ± 3.38	0.711
Medical history (n, %)			
Hypertension	21 (34.4)	24 (45.3)	0.237
Diabetes mellitus	6 (9.8)	9 (17.0)	0.260
Heart failure	14 (23.0)	13 (24.5)	0.843
Stroke	2 (3.3)	1 (1.9)	0.643
Coronary artery disease	0 (0.0)	5 (9.4)	0.014
Vascular disease	3 (4.9)	5 (9.4)	0.346
Pulmonary embolism	0 (0.0)	1 (1.9)	0.285
Hyperthyroidism	0 (0.0)	1 (1.9)	0.285
Renal dysfunction	0 (0.0)	1 (1.9)	0.285
Hepatic dysfunction	1 (1.8)	5 (9.4)	0.076
Current drinking	17 (27.9)	13 (24.5)	0.686
Current smoking	1 (1.6)	2 (3.8)	0.090
CHA2DS2-VASc score	1 (1–2.5)	2 (1–3)	0.001
Medication, n (%)			
Oral anticoagulant	1 (1.6)	21 (39.6)	< 0.001
Antiplatelet drug	1 (1.6)	11 (20.8)	0.001
ACEI/ARBs	14 (23.0)	14 (26.4)	0.668
CCB	7 (11.7)	18 (34.0)	0.004
Diuretic	0 (0.0)	8 (15.1)	0.002
Digoxin	0 (0.0)	10 (19.2)	< 0.001
Beta-blocker	3 (4.9)	28 (52.8)	< 0.001
Amiodarone	1 (1.6)	5 (9.4)	0.063

AF, atrial fibrillation; BMI, body mass index; CHA2DS2-VASc, congestive heart failure, hypertension, age ≥ 75, diabetes, stroke, vascular disease, age 65–74 and sex category(female); ACEI/ARBs, angiotensin-converting enzyme (ACE) inhibitors, angiotensin-receptor blockers; CCB, calcium channel blocker

### Accuracy and safety evaluation of wearable dynamic ECG recorders in 60 s in a supine position

The algorithm automatically determined the 60 s detection of the wearable dynamic ECG recorder for 47 cases of AF, 65 cases of non-AF, and 2 cases that could be judged (Table 2). The number of cases that were “unable to classify” was defined as “TPTN.” The diagnostic accuracy, sensitivity, and specificity using wearable dynamic ECG recorders were 96.49% (95% CI 91.03–98.92%), 92.45% (95% CI 81.64–97.52%), and 100% (95% CI 92.91–100%), respectively. The PPV was 100% (95% CI 91.32–100%), while NPV was 93.85% (95% CI 84.78–98.02%) (Table 3).

**Table 2:** Testing results of AF detection in participants and overall diagnostic performance of wearable dynamic ECG recorder in 60 s in a supine position

Wearable dynamic ECG recorder	Standard physician read ECG			
	AF	Sinus rhythm	Unclassified	N
AF	47	0	0	47
Non-AF	4	61	0	65
Unclassified	2	0	0	2
n	53	61	0	114

*ECG* electrocardiogram, *AF* atrial fibrillation

**Table 3:** Detailed diagnostic performance of the wearable dynamic ECG recorder for AF screening in a supine position

	Unable to unclassified as TPTN	Unable to unclassified as FPFN
Accuracy, %(95% CI)	96.49% (91.03–98.92%)	94.74% (88.76–97.80%)
Sensitivity, %(95% CI)	92.45% (81.64–97.52%)	88.68% (77.06–95.07%)
Specificity, %(95% CI)	100% (92.91–100%)	100% (92.91–100%)
PPV, %(95% CI)	100% (91.32–100%)	100% (90.98–100%)
NPV, %(95% CI)	93.85% (84.78–98.02%)	91.04% (81.48–96.16%)

*ECG* electrocardiogram, *AF* atrial fibrillation, *TP* true positives, *TN* true negatives, *FP* false positives, *FN* false negative, *PPV* positive predictive value, *NPV* negative predictive value

For cases, such as “unable to classify,” which were defined as “FPFN,” the diagnostic accuracy, sensitivity, and specificity using wearable dynamic ECG recorders were 94.74% (95% CI 88.76–97.80%), 88.68% (95% CI 77.06–95.07%), and 100% (95% CI 92.91–100%), respectively. The PPV was 100% (95% CI 90.98–100%), and NPV was 91.04% (95% CI 81.48–96.16%) (Table 3).

The medical devices tested in the study belong to category II medical devices with low-risk management levels. No adverse events or obvious device defects occurred within the entire investigation.

### Accuracy and safety evaluation of wearable dynamic ECG recorders in 60 s in an upright position

The algorithm automatically determined the 60 s detection of the wearable dynamic ECG recorder for 50 cases of AF, 63 cases of non-AF, and 1 case that could not be judged (Table 4). The number of cases that were “unable to classify” was defined as “TPTN.” The diagnostic accuracy, sensitivity, and specificity were 98.25% (95% CI% 93.43–99.91%), 96.23% (95% CI 86.51–99.69%), and 100% (95% CI 92.91–100%), respectively. The PPV was 100% (95% CI 91.63–100%), while NPV was 96.83% (95% CI 88.50–99.77%) (Table 5).

**Table 4:** Testing results of AF detection in participants and overall diagnostic performance of wearable dynamic ECG recorder in 60 s in an upright position

Wearable dynamic ECG recorder	Standard physician read ECG			
	AF	Sinus rhythm	Unclassified	N
AF	50	0	0	50
Non-AF	2	61	0	63
Unclassified	1	0	0	1
n	53	61	0	114

*ECG* electrocardiogram, *AF* atrial fibrillation

**Table 5:** Detailed diagnostic performance of the wearable dynamic ECG recorder for AF screening in an upright position

	Unable to unclassified as TPTN	Unable to unclassified as FPFN
Accuracy, %(95% CI)	98.25% (93.43–99.91%)	97.37% (92.21%, 99.44%)
Sensitivity, %(95% CI)	96.23% (86.51–99.69%)	94.34% (84.03–98.65%)
Specificity, %(95% CI)	100% (92.91–100%)	100% (92.91–100%)
PPV, %(95% CI)	100% (91.63–100%)	100% (91.48–100%)
NPV, %(95% CI)	96.83% (88.50–99.77%)	95.31% (86.57–98.92%)

*ECG* electrocardiogram, *AF* atrial fibrillation, *TP* true positives, *TN* true negatives, *FP* false positives, *FN* false negative, *PPV* positive predictive value, *NPV* negative predictive value

For cases such as “unable to classify,” which were defined as "FPFN,” the diagnostic accuracy, sensitivity, and specificity were 97.37% (95% CI 92.21–99.44%), 94.34% (95% CI 84.03–98.65%), and 100% (95% CI 92.91–100%), respectively. The PPV was 100% (95% CI 91.48–100%), while NPV was 95.31% (95% CI 86.57–98.92%) (Table 5). No adverse events or obvious device defects occurred within the entire investigation.

### Accuracy and safety evaluation of wearable dynamic ECG recorders in 60 s after climbed the third floor

Consistent results with those obtained for the upright position measurement were reported. There were 50 cases of AF, 63 cases of non-AF, and 1case that could not be judged (Table 6). No adverse events or obvious device defects occurred within the entire investigation (Table 7).

**Table 6:** Testing results of AF detection in participants and overall diagnostic performance of wearable dynamic ECG recorder in 60 s after climbed the third floor in an upright position

Wearable dynamic ECG recorder	Standard physician read ECG			
	AF	Sinus rhythm	Unclassified	N
AF	50	0	0	50
Non-AF	2	61	0	63
Unclassified	1	0	0	1
n	53	61	0	114

*ECG* electrocardiogram, *AF* atrial fibrillation

**Table 7:** Detailed diagnostic performance of the wearable dynamic ECG recorder for AF screening after climbed the third floor in an upright position

	Unable to unclassified as TPTN	Unable to unclassified as FPFN
Accuracy, %(95% CI)	98.25% (93.43–99.91%)	97.37%(92.21%, 99.44%)
Sensitivity, %(95% CI)	96.23% (86.51–99.69%)	94.34% (84.03–98.65%)
Specificity, %(95% CI)	100% (92.91–100%)	100% (92.91–100%)
PPV, %(95% CI)	100% (91.63–100%)	100% (91.48–100%)
NPV, %(95% CI)	96.83% (88.50–99.77%)	95.31% (86.57–98.92%)

*ECG* electrocardiogram, *AF* atrial fibrillation, *TP* true positives, *TN* true negatives, *FP* false positives, *FN* false negative, *PPV* positive predictive value, *NPV* negative predictive value

## Discussion

The accuracy of wearable dynamic ECG recorder for AF detection from sinus rhythm has herein been demonstrated in a small population trial setting. The finding provides a non-invasive, easy-to-use, and affordable tool to detect and discriminate AF in different positions and after exercises. To the best of our knowledge, the instrument used in this study is the first domestic tool applying an AI algorithm and a single-lead ECG wristband, which is user-friendly, easy to operate, and convenient to wear.

The wearable dynamic ECG recorder with an AI algorithm offers an accurate detection AF in different postures with 100% specificity and positive predictive value. When subjects are put to stand and tested after exercise, it becomes easier to detect the signal detection and make the correct diagnosis. In the present study, our AI algorithm has been fully trained and tested via large-scale real user data to ensure its reliability. AI is constructed using deep convolutional neural networks. The official data sensitivity and specificity of the test set were 93.36% and 99.75%, respectively. The single-lead ECG provides physicians with higher specificity and a clear review of the ECG records. At the same time, the wearable dynamic ECG recorder does not impact the daily activities of the subject. Our tested wristband did not require frequent communication with smartphones, consuming less power and increases the time of continuous data recording. Also, the wristband can stand on standby for 7 days on a fully charged battery.

The investigation provides data on the lying position, standing position, and exercise to simulate the test results of the tool in different states. Moreover, once the application detects AF, it promptly sends text messages to the wearer, related relatives, and a designated doctor assigned by Huami. The designated doctor would then diagnose and administer treatment to the patient.

Current evidence indicates that pulse palpation [17], 12-lead ECG [18], 24-h ECG Holter [19] and implanted cardioverter-defibrillator (ICD) [20] methods are some of the traditional methods for AF screening. However, these traditional methods are poised with challenges. With the advancement in technology, the present research has confirmed that smart devices, including mobile phones, handing devices, and wearable devices, can be employed for AF detection. McManus et al. [21] provided an algorithm connecting the root mean square of successive RR difference (RMSSD/mean) and Shannon entropy (ShE). The algorithm demonstrated excellent sensitivity of 96.2%, a specificity of 97.5%, and an accuracy of 96.8% for the beat-to-beat distinction of an irregular pulse during AF from sinus rhythm. Elsewhere, Svennberget et al. [22] described a handheld ECG recorder for intermittent ECG recordings, integrated with a mobile transmitter that sends 30-s ECG strip data to a database. In their analysis, repeated routine ECG examination over a long period potentially improved AF detection sensitivity to four times the number of cases diagnosed via routine ECG examination at a single time point.

The AF-SCREEN international collaboration [23], in 2017, confirmed the advantages of handheld ECG devices, particularly in providing a confirmable ECG trace required by the diagnostic guidelines for AF. Another mSToPS randomized clinical trial by Steinhubl et al. [24] provided evidence on the impact of a self-applied wearable ECG patch in AF detection. Further analysis demonstrated a higher new diagnosis rate of AF in the immediate group at 4 months than that of the delayed group (3.9% vs. 0.9%, the absolute difference was 3.0%, 95% CI 1.8–4.1%).

A recent technology of photo-plethysmography (PPG) application was mentioned for the detection of AF. The sensitivity and specificity of the PPG algorithm for AF detection were 97–100% and 92–94%, respectively [25]. Furthermore, conclusions of Heart-Study-MAFA II [26] showed higher sensitivity and specificity of the PPG method in AF screening. The sensitivity, specificity, and a positive predictive value achieved with their method were 100%, 99%, and 91.6%, respectively. Their data were collected in a supine position, therefore, does not reflect the actual situation for home screening in which the movement has a more significant influence on the PPG signal. Of note, interference caused by movement should be avoided between single-lead ECG and the PPG technology to improve accuracy. Also, there is a tendency to use single-lead ECG recordings in several patients with paroxysmal AF since single-lead ECG recordings with mobile phones or wristbands for AF detection are clinically superior to PPG signals.

Emerging data shows that early intervention and qualifiable risk factor control can reduce the incidence of AF. In this view, a wearing dynamic ECG recorder may provide an essential tool for patients with or at risk to detect AF. This approach allows timely detection and management of AF before adverse health consequences such as ischemic stroke or heart failure occur. With the increase of health awareness, wearable monitoring technology is gaining attention in health, allowing patients to comfortably manage symptoms from their places of residence. The wearable dynamic ECG recorder is a feasible and accurate tool that allows users to monitor and track their ECG recordings and share them with the attending physicians.

There are a few limitations to the study. Firstly, the ECG monitoring was performed in asymptomatic participants and not in subjects with symptoms. Secondly, the instruments were only used for discriminating between AF and sinus rhythm yet the wearable dynamic ECG recorder could detect other arrhythmias, including premature beats, atrial tachyarrhythmias, and atrial flutter. In our future study, new algorithms will be integrated to assist the identification and distinction between sinus arrhythmias and various arrhythmia forms. Lastly, owing to the sample size was relatively small, more extensive testing would be warranted in the future to verify the results.

## Conclusions

The broadly accessible wearable dynamic ECG recorder with an AI algorithm can detect AF and analyze heart rhythms at different postures and after exercises. This approach may provide a user-friendly and accurate method to discriminate AF and timely detect asymptomatic AF patients or those at risk of AF. More extensive clinical trials are needed to assess the effectiveness of this technique in monitoring and early diagnosis of AF recurrence.

## Data Availability

Datasets used and/or analyzed in this study are de-identified and available from the corresponding author on reasonable request.

## Abbreviations

AF: Atrial fibrillation
ECG: Electrocardiogram
AI: Artificial intelligence
PPV: Positive predictive value
NPV: Negative predictive value
AE: Adverse events
SAE: Serious adverse events
SD: Standard deviation
TP: True positives
TN: True negatives
FP: False positives
FN: False negative
CCB: Calcium channel blockers
ICD: Implanted cardioverter-defibrillator
She: Shannon entropy
PPG: Photo-plethysmography
